# Virulome and genome analyses identify associations between antimicrobial resistance genes and virulence factors in highly drug-resistant *Escherichia coli* isolated from veal calves

**DOI:** 10.1371/journal.pone.0265445

**Published:** 2022-03-17

**Authors:** Bradd J. Haley, Seon Woo Kim, Serajus Salaheen, Ernest Hovingh, Jo Ann S. Van Kessel

**Affiliations:** 1 Environmental Microbial and Food Safety Laboratory, Beltsville Agricultural Research Center, Agricultural Research Service, United States Department of Agriculture, Beltsville, MD, United States of America; 2 Department of Veterinary and Biomedical Sciences, Pennsylvania State University, University Park, PA, United States of America; Tianjin University, CHINA

## Abstract

Food animals are known reservoirs of multidrug-resistant (MDR) *Escherichia coli*, but information regarding the factors influencing colonization by these organisms is lacking. Here we report the genomic analysis of 66 MDR *E*. *coli* isolates from non-redundant veal calf fecal samples. Genes conferring resistance to aminoglycosides, β-lactams, sulfonamides, and tetracyclines were the most frequent antimicrobial resistance genes (ARGs) detected and included those that confer resistance to clinically significant antibiotics (*bla*_CMY-2_, *bla*_CTX-M_, *mph(A)*, *erm(B)*, *aac(6’)Ib-cr*, and *qnrS1*). Co-occurrence analyses indicated that multiple ARGs significantly co-occurred with each other, and with metal and biocide resistance genes (MRGs and BRGs). Genomic analysis also indicated that the MDR *E*. *coli* isolated from veal calves were highly diverse. The most frequently detected genotype was phylogroup A-ST Cplx 10. A high percentage of isolates (50%) were identified as sequence types that are the causative agents of extra-intestinal infections (ExPECs), such as ST69, ST410, ST117, ST88, ST617, ST648, ST10, ST58, and ST167, and an appreciable number of these isolates encoded virulence factors involved in the colonization and infection of the human urinary tract. There was a significant difference in the presence of multiple accessory virulence factors (VFs) between MDR and susceptible strains. VFs associated with enterohemorrhagic infections, such as *stx*, *tir*, and *eae*, were more likely to be harbored by antimicrobial-susceptible strains, while factors associated with extraintestinal infections such as the *sit* system, aerobactin, and *pap* fimbriae genes were more likely to be encoded in resistant strains. A comparative analysis of SNPs between strains indicated that several closely related strains were recovered from animals on different farms indicating the potential for resistant strains to circulate among farms. These results indicate that veal calves are a reservoir for a diverse group of MDR *E*. *coli* that harbor various resistance genes and virulence factors associated with human infections. Evidence of co-occurrence of ARGs with MRGs, BRGs, and iron-scavenging genes (*sit* and aerobactin) may lead to management strategies for reducing colonization of resistant bacteria in the calf gut.

## Introduction

*Escherichia coli* are Gram-negative facultative anaerobes that are commensal members of the bovine gut as well as frequent members of environmental (non-animal) communities. Most *E*. *coli* are non-pathogenic, but a small subset has been linked to a range of mild to severe human diseases. These typically include self-limiting gastrointestinal (GI) infections as well as extra-intestinal infections such as bladder/urinary tract infections (UTIs), prostatitis, wound infections, pneumonia, sepsis, and meningitis in newborn babies. Infections are primarily caused by strains that carry various suites of virulence factors (VFs), but opportunistic infections can be caused by any strain, even those lacking major VFs. *E*. *coli* causes a significant number of GI infections annually in the United States and is responsible for 80% of UTIs [1–3]. Treatments for non-Shiga-toxigenic infections typically involve antimicrobial therapy, but pathogenic and non-pathogenic *E*. *coli* can be resistant to these drugs; some are multidrug-resistant (MDR) and can cause difficult-to-treat infections in humans and animals. Further, the *E*. *coli* population can serve as a reservoir of resistance genes that can transfer from commensal to pathogenic strains, or to other pathogenic organisms, such as *Salmonella enterica* [4–6].

Antimicrobial-resistant infections are an on-going human and animal health threat on a global scale, causing an extremely high, but not well-quantified, number of medical complications and fatalities each year [7–9]. Like antimicrobial-susceptible organisms, infections caused by resistant organisms can be nosocomial, community-acquired, waterborne, or foodborne. Foodborne and waterborne antimicrobial-resistant *E*. *coli* infections typically occur from fecal contamination of produce, meat, milk, eggs, and surface and drinking waters; community-acquired resistant *E*. *coli* infections, although transmitted differently than foodborne and waterborne infections, can be caused by strains that have a natural food animal host reservoir, such as poultry and cattle.

Beef cattle, dairy cows, and dairy calves are well documented reservoirs of antimicrobial-resistant bacteria and pathogens, but antimicrobial resistance carriage in veal calves remains understudied [10–13]. Calves raised for veal are usually the male calves from dairy herds. In the United States, milk is a major component of their diet until they are 16 to 18 weeks of age. About 15% of marketed veal calves are “bob veal” which are sold from birth up to three weeks of age. Recent studies have shown that dairy calf feces are a significant source of resistant bacteria and typically harbor a different suite of antimicrobial resistance genes (ARGs) and a greater concentration of ARGs than older lactating and dry cows [10–14]. However, the genetic mechanisms or management practices responsible for these age-related differences in resistance carriage remain unknown. Further, the characteristics of resistant bacteria shed by veal calves, which are raised under significantly different management practices than replacement dairy calves, have not been adequately studied. The aim of this study was to comprehensively evaluate the genomic characteristics, virulence profiles, and ARGs in MDR *E*. *coli* collected from veal calf feces, as well as the genomic features that co-occur with these genes and may influence resistance carriage in the veal calf gut. We further compared the genomic distance between isolates to investigate the relatedness of isolates collected from animals on different farms.

## Materials and methods

In total, 66 confirmed MDR *E*. *coli* isolates collected from veal calves during a previous study [15] were selected for whole genome sequencing (WGS). Simultaneously, a subset of 19 pansusceptible isolates from the same study were selected as comparators for the MDR genome analyses. *E*. *coli* isolates were recovered from feces collected directly from individual calves on 12 farms at two time points; once immediately after arriving at the farm (typically ~ 1 week of age) and then again immediately prior to slaughter (at or around 24 weeks of age) [15]. In order to reduce within-individual animal bias and prevent the selection of within-sample enrichment of clonal isolates, only one isolate per resistance group (MDR or susceptible) was selected for each animal from each time point. Antibiotic-susceptible strains were randomly selected from unique animals, and MDR isolates that were resistant to the most classes of antibiotics were selected to evaluate highly drug-resistant isolates. Multidrug-resistant isolates were considered those that were resistant to at least three classes of antibiotics. Isolates were not selected from all animals due to feasibility. Veal calf management protocols were not made available. Sampling procedures were approved by the Pennsylvania State University institutional animal care and use committee (IACUC, protocol number 42381–1).

*E*. *coli* isolates were grown overnight at 37°C in Luria-Bertani broth (BD Diagnostics, Sparks, MD), and DNA was extracted using the QIAmp DNA Mini kit (Qiagen, Hilden, Germany). For shotgun genome sequencing, DNA libraries were constructed using the Nextera XT chemistry (Illumina, La Jolla, CA) and these were sequenced on the NextSeq 500 platform (2 x 151 bp paired-end reads) (Illumina). Sequencing reads were subsequently trimmed and curated for quality, length and contaminants using Trimmomatic (LEADING:20 TRAILING:20 SLIDINGWINDOW:4:20 MINLEN:36) [16] and DeconSeq to remove phiX reads (NCBI accession: NC_001422) [17]. Reads were assembled using SPAdes V. 3.14.1 with the–careful option to reduce the number of mismatches and indels [18]. Genome sequencing data and metadata have been deposited at NCBI under BioProject ID PRJNA664052.

Assembled genomes were evaluated for Sequence Type (ST) assignment [19], plasmid replicons [20], and antimicrobial resistance genes [21] under default settings using the Center for Genomic Epidemiology webserver (http://www.genomicepidemiology.org/). Phylogroups were determined using the ClermonTyping and EZClermont schemes [22, 23]. Virulence factors were identified using standalone BLASTN with a minimum 80% nucleotide sequence similarity across a minimum of 80% of the length of the reference VF gene selected from a database of known *E*. *coli* virulence genes [24]. Metal and biocide resistance genes (MRGs and BRGs) were similarly identified with BLASTN using *Enterobacteriaceae* reference nucleotide sequences from the BacMet database [25].

To visualize the differences in the virulomes (the set of virulence factors that are involved in virulence of a bacterium) of MDR and susceptible genomes, a nonmetric multidimensional scaling (NMDS) analysis using the Jaccard distance metric was inferred using the vegan package, followed by an analysis of similarities (ANOSIM) of the virulomes of these two groups using vegan in R [26]. The enrichment of VFs in MDR or susceptible isolates was evaluated using a two-tailed Fisher’s exact test (fisher.test command in R) followed by a correction for multiple comparisons [27] using the command p.adjust in the stats package (method = “fdr”) and the qvalue command (default commands) in the qvalue package in R. These corrected P-values are written as q-values. For this analysis, q < 0.05 (false discovery rate = 5%) was considered statistically significant. Analyses to determine the co-occurrence between VFs and ARG/MRG/BRGs, and plasmid replicons and ARG/MRG/BRGs were conducted with the cooccur command with the parameter thresh = TRUE to remove cooccurrences that are not expected to occur more than once, in the cooccur package in R [28]. The presence of ARGs and VFs in the isolates was visualized using ForceAtlas2 algorithm on an interactive network inference, Gephi version 0.9.1 (scaling 10, edge weight influence 1) [29, 30]. ForceAtlas2 is a force-directed algorithm used for network spatialization where nodes repulse each other like charged particles while edges attract their nodes [30]. These parameters were chosen for clarity of the nodes in the network. The edges (curves) in the network links a gene to the host isolate.

Core genome single nucleotide polymorphisms (SNPs) were identified by aligning the 85 *E*. *coli* genomes used in this study with 118 publicly available *Escherichia* genomes representing the eight major phylogenetic groups (A, B1, B2, C, D, E, F, and G), *E*. *coli* Cryptic Clades, and the near neighbors *E*. *fergussoni* and *E*. *albertii* using the Harvest package [31]. ParSNP within the Harvest package was run with the following parameters, -c (to force inclusion of all genomes in the analysis) and -x (to enable recombination filtering), and the complete chromosome of *E*. *coli* K-12 substr. MG1655 (NCBI accession: NC_000913.3) as the reference genome (-r). These SNPs were then used to infer a maximum likelihood tree with 1000 bootstrap replicates under default settings using the Randomized Axelerated Maximum Likelihood program (RAxML) [32]. To interrogate the relatedness of isolates collected from different farms, high quality SNPs (hqSNPs) were identified by aligning the cleaned and curated reads of presumptive related genomes to the *E*. *coli* K-12 MG1655 genome and retaining those SNPs that have a minimum of 10X sequencing read coverage by using the program Lyve-SET [33].

## Results

**Fig 1:**
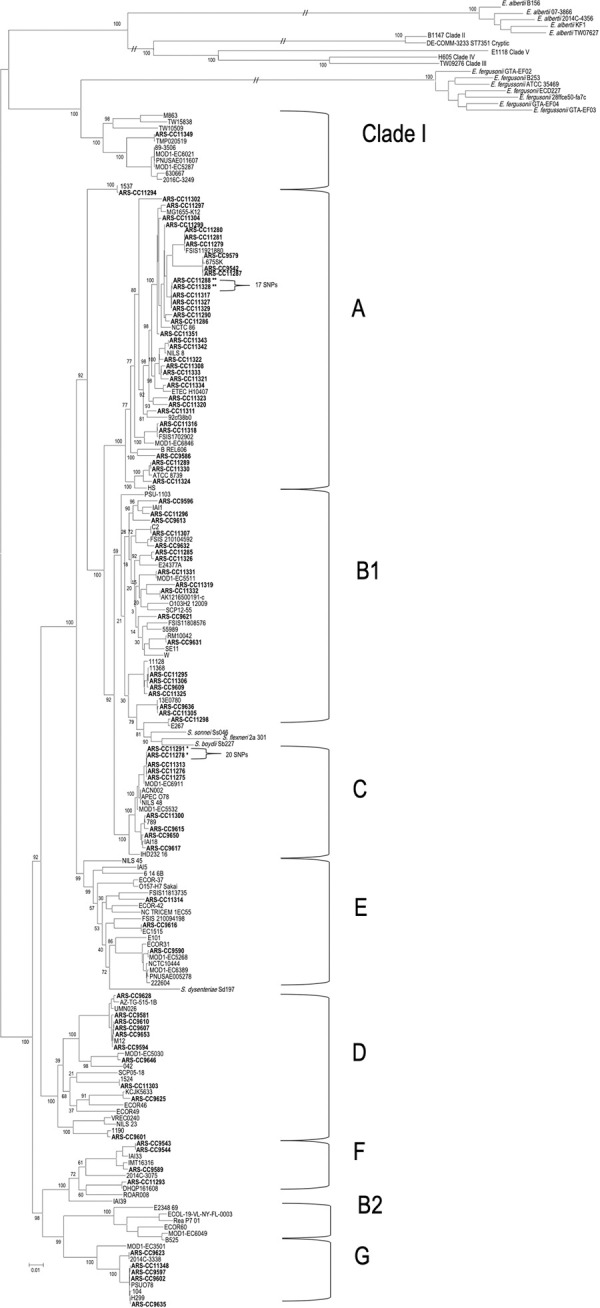
Maximum likelihood tree showing the inferred phylogeny of strains analyzed in this study (in bold with “ARS-CC” prefix) along with previously characterized strains from each phylogroup, cryptic clade, and *E*. *albertii*, and *E*. *fergusonii*, with 1000 bootstrap replicates. Brackets on right show phylogroups. The two inner brackets show the SNP differences between closely related strains. The bar on the bottom left shows substitutions per site.

Among the MDR isolates, phylogroups A, B1, D, C, F, G, E were identified 29, 9, 9, 8, 4, 4, and 2 times, respectively, with a single isolate identified as a member of Clade I (Fig 1; Table 1). None of the isolates were identified as phylogroup B2. In total, 33 STs were identified, and the most common were ST10 (7 isolates, 10.6% of isolates), ST69 (6, 9.1%), ST744 (5, 7.6%), and ST410 (5, 7.6%) (Table 1). There was a total of 12 ST complexes (ST Cplx), which include closely related STs differing by only a few alleles. The most frequently detected ST complexes were the ST10 Cplx (22 isolates, 33%), ST23 Cplx (9 isolates, 13.6%), ST69 Cplx (6 isolates, 9.1%), and ST648 Cplx (3 isolates, 4.5%). There were 16 isolates (24.2%) that were not assigned to any ST Cplx (Table 1).

**Table 1:** Characteristics of MDR and susceptible strains. Columns show farm from which the isolate was collected, MLST, ST Cplx, antibiotic resistance, metal resistance, and biocide resistance genes.

Isolate	Farm	MLST	ST Cplx	Phylogroup	Antibiotic Resistance Genes (ARG)	Antibiotic Resistance-Conferring Point Mutations	Metal Resistance Genes (MRG)	Biocide Resistance Genes (BRG)	Plasmid Replicons
ARS-CC11286	K	10	10	A	*aadA5*, *bla*, *blaTEM-1B*, *dfrA17*, *erm(B)*, *floR*, *mph(A)*, *aph(3’’)-Ib*, *aph(6)-Id*, *sul1*, *sul2*, *tet(A)*, *tet(B)*	*gyrA* (p.S83L), *gyrA* (p.D87N)	*merA*	*qacEΔ1*, *sugE*	IncFII, IncFIB(pB171), IncX1
ARS-CC11297	D	10	10	A	*aadA1*, *aadA2*, *aph(3’)-Ic*, *blaTEM-1C*, *cmlA1*, *dfrA12*, *floR*, *aph(3’’)-Ib*, *aph(6)-Id*, *sul2*, *sul3*, *tet(A)*, *tet(M)*		* *	*sugE*	IncFII, IncN, IncX1, p0111, IncQ1, ColRNAI
ARS-CC11299	D	10	10	A	*aadA5*, *blaCTX-M-15*, *blaTEM-1A*, *dfrA17*, *erm(B)*, *floR*, *mph(A)*, *aph(3’’)-Ib*, *aph(6)-Id*, *sul1*, *sul2*, *tet(A)*, *tet(B)*	*gyrA* (p.S83L), *gyrA* (p.D87N)	*silABC*	*qacEΔ1*, *sugE*	IncFIB(pB171), IncFII, IncI1, IncFIB(K), ColRNAI
ARS-CC11321	A	10	10	A	*aac(3)-VIa*, *aadA1*, *aadA5*, *aph(3’)-Ia*, *blaTEM-1B*, *floR*, *aph(3’’)-Ib*, *aph(6)-Id*, *sul1*, *sul2*, *tet(A)*		* *	* *	IncFIB(K), IncFIB(pB171), IncX1, IncA/C2
ARS-CC11322	D	10	10	A	*aadA1*, *aph(3’)-Ic*, *blaCMY-2*, *blaTEM-1B*, *dfrA1*, *floR*, *aph(3’’)-Ib*, *aph(6)-Id*, *sul2*, *tet(A)*, *tet(M)*		* *	* *	IncN, IncFII(pCoo), IncY, IncA/C2
ARS-CC11333	K	10	10	A	*aadA2*, *blaCMY-2*, *dfrA12*, *floR*, *mph(A)*, *aph(3’’)-Ib*, *aph(6)-Id*, *sul1*, *sul2*, *tet(A)*, *tet(M)*		* *	*qacEΔ1*, *sugE*, *sugE1*	IncA/C2, ColpVC, ColRNAI
ARS-CC11351	M	10	10	A	*blaCMY-2*, *dfrA17*, *floR*, *aph(3’’)-Ib*, *aph(6)-Id*, *sul2*, *tet(A)*, *tet(M)*		* *	*qacEΔ1*, *sugE*, *sugE1*	p0111, IncA/C2
ARS-CC11279	E	44	10	A	*aadA5*, *blaCTX-M-15*, *dfrA17*, *mph(A)*, *aph(3’’)-Ib*, *aph(6)-Id*, *sul1*, *sul2*, *tet(B)*	*gyrA* (p.S83L), *gyrA* (p.D87N)	* *	*qacEΔ1*, *sugE*	IncFII, IncI1, IncFIB(AP001918), IncFIA, Col(MGD2), Col(MG828), ColRNAI
ARS-CC11280	F	44	10	A	*aadA5*, *blaCTX-M-15*, *dfrA17*, *mph(A)*, *sul1*, *tet(B)*	*gyrA* (p.S83L), *gyrA* (p.D87N)	* *	*qacEΔ1*, *sugE*	IncFIA, IncI1, IncFIB(AP001918), IncFII, Col(MGD2), Col(MG828), IncB/O/K/Z, ColRNAI
ARS-CC11281	E	44	10	A	*aadA5*, *blaCTX-M-15*, *dfrA17*, *mph(A)*, *sul1*, *tet(B)*	*gyrA* (p.S83L), *gyrA* (p.D87N)	* *	*qacEΔ1*, *sugE*	IncFII(pRSB107), IncFII, IncI1, IncFIB(AP001918), IncFIA, Col(MGD2), ColRNAI, IncB/O/K/Z, Col(MG828)
ARS-CC11334	L	48	10	A	*aadA1*, *aadA2*, *aph(3’)-Ia*, *blaCMY-2*, *blaTEM-1B*, *cmlA1*, *dfrA12*, *floR*, *lnu(F)*, *sul2*, *sul3*, *tet(A)*		* *	*sugE*, *sugE1*	IncFIC(FII), IncI1, IncFIB(AP001918), IncX1, ColRNAI
ARS-CC11290	J	167	10	A	*aac(6’)Ib-cr*, *aadA1*, *aadA5*, *blaCTX-M-15*, *blaOXA-1*, *catB3*, *dfrA17*, *lnu(F)*, *mph(A)*, *sul1*, *tet(A)*	*gyrA* (p.S83L), *gyrA* (p.D87N)	* *	*qacEΔ1*, *sugE*	IncFII, IncFIB(AP001918), IncFIA, ColRNAI
ARS-CC11287	G	617	10	A	*aadA1*, *aadA2*, *aph(3’)-Ia*, *blaCTX-M-55*, *blaTEM-1B*, *dfrA12*, *floR*, *lnu(F)*, *sul3*, *tet(A)*, *tet(M)*	*gyrA* (p.S83L), *gyrA* (p.D87N)	*merA*, *silAB*, *pcoABCDRSE*	*sugE*	IncFIB(AP001918), IncFII, IncFIC(FII), IncX1
ARS-CC9542	B	617	10	A	*aac(3)-IIa*, *aac(6’)Ib-cr*, *aadA5*, *blaCTX-M-1*, *blaOXA-1*, *catB3*, *dfrA17*, *mph(A)*, *aph(3’’)-Ib*, *aph(6)-Id*, *sul1*, *sul2*, *tet(B)*	*gyrA* (p.S83L), *gyrA* (p.D87N)	* *	*qacEΔ1*, *sugE*	IncFII, IncFIB(AP001918), IncFIA, IncN, IncX1, ColRNAI, Col(MG828)
ARS-CC9579	B	617	10	A	*aac(6’)Ib-cr*, *aadA1*, *aadA5*, *aph(3’)-Ic*, *blaCTX-M-15*, *blaOXA-1*, *catA1*, *catB3*, *dfrA17*, *floR*, *aph(3’’)-Ib*, *aph(6)-Id*, *sul1*, *sul2*, *tet(A)*, *tet(B)*	*gyrA* (p.S83L), *gyrA* (p.D87N)	* *	*qacEΔ1*, *sugE*	IncFII, IncFIB(Mar), IncFIB(AP001918), IncFIA, IncHI1B, IncA/C2, ColRNAI
ARS-CC11288	G	744	10	A	*aadA5*, *blaCTX-M-55*, *blaTEM-1B*, *catA1*, *dfrA17*, *mph(A)*, *aph(3’’)-Ib*, *aph(6)-Id*, *sul1*, *sul2*, *tet(B)*	*parC* (p.A56T), *gyrA* (p.S83L), *gyrA* (p.D87N)	*merA*	*qacEΔ1*, *sugE*	IncFII, IncFIB(AP001918), IncQ1
ARS-CC11317	J	744	10	A	*aac(3)-VIa*, *aadA1*, *aadA2*, *aph(3’)-Ia*, *blaCMY-2*, *blaTEM-1B*, *catA1*, *dfrA12*, *floR*, *mph(A)*, *aph(3’’)-Ib*, *aph(6)-Id*, *sul1*, *sul2*, *tet(A)*, *tet(B)*, *tet(M)*	*parC* (p.A56T), *gyrA* (p.S83L), *gyrA* (p.D87N)	* *	* *	IncHI2, IncHI2A, IncFII, IncI1, TrfA, IncQ1, IncA/C2
ARS-CC11327	B	744	10	A	*aadA5*, *blaCMY-2*, *blaTEM-1B*, *catA1*, *dfrA17*, *mph(A)*, *aph(3’’)-Ib*, *aph(6)-Id*, *sul1*, *sul2*, *tet(B)*	*parC* (p.A56T), *gyrA* (p.S83L), *gyrA* (p.D87N)	*merA*	*qacEΔ1*, *sugE*, *sugE1*	IncI1, IncQ1
ARS-CC11328	B	744	10	A	*aadA5*, *blaCTX-M-55*, *blaTEM-1B*, *catA1*, *dfrA17*, *mph(A)*, *aph(3’’)-Ib*, *aph(6)-Id*, *sul1*, *sul2*, *tet(B)*	*gyrA* (p.S83L), *parC* (p.A56T)	*merA*	*qacEΔ1*, *sugE*	IncFII, IncFIB(AP001918), IncQ1
ARS-CC11329	I	744	10	A	*aadA1*, *aadA2*, *aph(3’)-Ia*, *blaCTX-M-15*, *blaTEM-1B*, *cmlA1*, *dfrA12*, *mef(B)*, *mph(A)*, *aph(3’’)-Ib*, *aph(6)-Id*, *sul1*, *sul2*, *sul3*, *tet(B)*, *tet(M)*	*gyrA* (p.S83L), *parC* (p.A56T)	*merA*	*qacEΔ1*, *sugE*	IncFIC(FII), IncFII, IncFIB(AP001918), IncQ1
ARS-CC11311	H	993	10	A	*aadA2*, *aph(3’)-Ia*, *blaTEM-1B*, *dfrA12*, *floR*, *mph(A)*, *aph(3’’)-Ib*, *aph(6)-Id*, *sul1*, *sul2*, *tet(A)*, *tet(B)*		* *	* *	IncFIC(FII), IncFIB(AP001918), IncX1, ColRNAI, IncA/C2, Col(MG828)
ARS-CC11323	F	1721	10	A	*aadA2*, *aph(3’)-Ia*, *blaTEM-1B*, *catA1*, *dfrA12*, *floR*, *mph(A)*, *aph(3’’)-Ib*, *aph(6)-Id*, *sul1*, *sul2*, *tet(A)*, *tet(M)*		*merA*, *silABC*,*arsRDABC*, *pcoABCDRSE*	*qacEΔ1*, *sugE*	IncFIA(HI1), IncFII, IncFIB(K), IncFII(Yp), IncX1, IncA/C2, ColRNAI
ARS-CC11302	F	1114	165	A	*aadA2*, *aph(3’)-Ia*, *blaCMY-2*, *catA1*, *dfrA12*, *floR*, *mph(A)*, *aph(3’’)-Ib*, *aph(6)-Id*, *sul1*, *sul2*, *tet(A)*, *tet(M)*	*gyrA* (p.S83L), *gyrA* (p.D87N)	* *	* *	IncFII, p0111, ColRNAI, Col(MG828)
ARS-CC11289	I	2325	467	A	*aadA1*, *aadA2*, *aph(3’)-Ia*, *blaCTX-M-27*, *blaTEM-1B*, *cmlA1*, *dfrA12*, *erm(B)*, *floR*, *lnu(F)*, *sul2*, *sul3*, *tet(A)*, *tet(M)*	*gyrA* (p.S83L), *gyrA* (p.D87N)	* *	*sugE*	IncFIC(FII), IncI1, IncFIB(AP001918), IncX1, ColRNAI, Col(MG828)
ARS-CC11330	I	2325	467	A	*aadA1*, *aadA2*, *aph(3’)-Ia*, *blaCTX-M-27*, *blaTEM-1B*, *cmlA1*, *dfrA12*, *erm(B)*, *floR*, *lnu(F)*, *sul2*, *sul3*, *tet(A)*, *tet(M)*	*gyrA* (p.S83L), *gyrA* (p.D87N)	* *	*sugE*	IncFIC(FII), IncI1, IncFIB(AP001918), IncX1, ColRNAI, Col(MG828)
ARS-CC11316	J	540		A	*aadA1*, *aph(3’)-Ic*, *blaTEM-1B*, *dfrA1*, *floR*, *aph(3’’)-Ib*, *aph(6)-Id*, *sul1*, *sul2*, *tet(A)*, *tet(M)*	*gyrA* (p.S83L)	* *	* *	IncFII, IncFIA, Col(MG828), ColRNAI, IncA/C2, IncX1, Col8282, IncQ1
ARS-CC11318	K	540		A	*aadA5*, *aph(3’)-Ia*, *blaCMY-2*, *blaTEM-1B*, *floR*, *aph(3’’)-Ib*, *aph(6)-Id*, *sul1*, *sul2*, *tet(A)*		* *	* *	p0111, IncA/C2
ARS-CC9586	B	1112		A	*aph(3’)-Ia*, *blaCMY-2*, *blaTEM-1B*, *floR*, *aph(3’’)-Ib*, *aph(6)-Id*, *sul2*, *tet(A)*, *tet(B)*, *tet(C)*, *tet(M)*		* *	*sugE*, *sugE1*	IncFII(pSE11), IncI1, IncFIB(AP001918), IncA/C2
ARS-CC11320	M	1564		A	*aadA5*, *aph(3’)-Ia*, *blaCMY-2*, *dfrA17*, *floR*, *aph(3’’)-Ib*, *aph(6)-Id*, *sul1*, *sul2*, *tet(A)*, *tet(M)*		* *	* *	IncFIB(K), p0111, IncA/C2
ARS-CC11331	J	345	23	B1	*aadA12*, *aadA2*, *blaTEM-1B*, *dfrA23*, *floR*, *aph(3’’)-Ib*, *aph(6)-Id*, *sul1*, *sul2*, *tet(A)*, *tet(M)*		*merA*	*qacEΔ1*, *sugE*	IncFIB(AP001918), IncY, IncQ1
ARS-CC9632	C	58	155	B1	*aph(3’)-Ic*, *aph(3’’)-Ib*, *aph(6)-Id*, *sul2*, *tet(B)*		* *	*sugE*	IncFII(pRSB107), IncFIA, IncFIB(AP001918), IncFII(pCoo), IncX1, IncI2, IncX4, ColRNAI
ARS-CC11285	I	448	448	B1	*aph(3’)-Ia*, *blaCTX-M-27*, *blaTEM-1B*, *dfrA7*, *rmtE*, *aph(3’’)-Ib*, *aph(6)-Id*, *sul1*, *sul2*, *tet(M)*	*gyrA* (p.S83L), *gyrA* (p.D87N)	* *	*qacEΔ1*, *sugE*	IncHI2, IncFII(pCoo), IncHI2A, IncFII, IncFIB(AP001918), IncFIA, TrfA, ColRNAI, Col(MG828), Col156, IncQ1, IncB/O/K/Z
ARS-CC11326	B	448	448	B1	*blaCMY-2*, *dfrA8*, *aph(3’’)-Ib*, *aph(6)-Id*, *sul2*, *tet(A)*, *tet(M)*		* *	*sugE*	IncFII, IncI1, IncQ1
ARS-CC9596	C	947	469	B1	*aac(3)-IId*, *aadA5*, *aph(3’)-Ic*, *blaCMY-2*, *blaTEM-1B*, *dfrA17*, *aph(3’’)-Ib*, *aph(6)-Id*, *sul1*, *sul2*, *tet(B)*		* *	*qacEΔ1*, *sugE*, *sugE1*	IncFIA, IncFIB(AP001918), IncFII(pCoo),
ARS-CC11319	K	224		B1	*aadA1*, *aadA2*, *blaCMY-2*, *blaTEM-1B*, *cmlA1*, *dfrA12*, *floR*, *lnu(F)*, *mph(A)*, *aph(3’’)-Ib*, *aph(6)-Id*, *sul1*, *sul2*, *sul3*, *tet(A)*, *tet(B)*, *tet(M)*	*gyrA* (p.S83L), *gyrA* (p.D87N)	* *	* *	IncI1, IncX1, IncY, IncA/C2
ARS-CC11298	D	2436		B1	*aac(3)-VIa*, *aadA1*, *aadA2*, *aph(3’)-Ia*, *blaTEM-1B*, *dfrA12*, *floR*, *aph(3’’)-Ib*, *aph(6)-Id*, *sul1*, *sul2*, *tet(A)*, *tet(M)*		*merA*	*qacEΔ1*, *sugE*	IncFII, IncFIB(AP001918), IncQ1, ColRNAI
ARS-CC11307	G	2522		B1	*aph(3’’)-Ib*, *aph(6)-Id*, *sul2*, *tet(B)*		* *	* *	
ARS-CC11332	K	6345		B1	*aadA2*, *blaCMY-2*, *dfrA12*, *floR*, *aph(3’’)-Ib*, *aph(6)-Id*, *sul1*, *sul2*, *tet(A)*		* *	*qacEΔ1*, *sugE*, *sugE1*	IncFII
ARS-CC11300	E	88	23	C	*aadA2*, *aph(3’)-Ia*, *blaCMY-2*, *dfrA12*, *floR*, *mph(A)*, *aph(3’’)-Ib*, *aph(6)-Id*, *sul1*, *sul2*, *tet(A)*, *tet(M)*	*ampC*-promoter (g.-42C>T)	* *	* *	IncA/C2
ARS-CC9615	B	88	23	C	*aadA1*, *blaTEM-1B*, *dfrA1*, *aph(3’’)-Ib*, *aph(6)-Id*, *sul1*, *sul2*, *tet(A)*		*merA*	*qacEΔ1*, *sugE*	IncFII, IncFIB(AP001918), Col(MGD2), IncQ1, Col156, IncB/O/K/Z, ColRNAI
ARS-CC9650	C	88	23	C	*aadA1*, *aadA2*, *aph(3’)-Ic*, *blaCMY-2*, *blaTEM-1B*, *dfrA1*, *floR*, *mph(A)*, *aph(3’’)-Ib*, *aph(6)-Id*, *sul1*, *sul2*, *tet(A)*, *tet(B)*, *tet(M)*	*ampC*-promoter (g.-42C>T)	* *	*qacEΔ1*, *sugE*, *sugE1*	IncFIA, IncFIB(AP001918), IncFII(pCoo), IncA/C2
ARS-CC11275	D	410	23	C	*aac(3)-IIa*, *aadA5*, *blaCTX-M-15*, *dfrA17*, *mph(A)*, *sul1*, *tet(B)*	*gyrA* (p.S83L), *gyrA* (p.D87N)	*silABC*, *pcoABCDRSE*	*qacEΔ1*, *sugE*	IncFIA, IncFIB(AP001918), IncFII, IncQ1
ARS-CC11276	D	410	23	C	*aac(3)-IIa*, *aadA5*, *blaCTX-M-15*, *dfrA17*, *mph(A)*, *sul1*, *tet(B)*	*gyrA* (p.S83L), *gyrA* (p.D87N)	*silABC*, *pcoABCDRSE*	*qacEΔ1*, *sugE*	IncFII(pRSB107), IncFII, IncFIB(AP001918), IncFIA, IncQ1
ARS-CC11278	E	410	23	C	*aadA1*, *aadA5*, *aadB*, *blaCTX-M-15*, *blaTEM-1C*, *cmlA1*, *dfrA17*, *mph(A)*, *sul1*, *tet(A)*	*gyrA* (p.S83L), *gyrA* (p.D87N)	*silABC*, *pcoABCDRSE*	*qacEΔ1*, *sugE*	IncFII, IncFIB(AP001918), IncFIA, IncX1, ColRNAI
ARS-CC11291	H	410	23	C	*aadA1*, *aadA5*, *aadB*, *blaCTX-M-15*, *blaTEM-1C*, *cmlA1*, *dfrA17*, *mph(A)*, *sul1*, *tet(A)*	*gyrA* (p.S83L), *gyrA* (p.D87N)	*silABC*, *pcoABCDRSE*	*qacEΔ1*, *sugE*	IncFIA, IncFIB(AP001918), IncFII,
ARS-CC11313	H	410	23	C	*aadA1*, *aadA5*, *aadB*, *blaCTX-M-15*, *blaTEM-1C*, *cmlA1*, *dfrA17*, *mph(A)*, *sul1*, *tet(A)*	*gyrA* (p.S83L), *gyrA* (p.D87N)	* *	* *	IncFII, IncFIB(AP001918), IncFIA,
ARS-CC9646	B	925	31	D	*aadA1*, *aph(3’)-Ic*, *blaCMY-2*, *blaTEM-1D*, *dfrA1*, *sul1*, *tet(A)*		*merA*	*qacEΔ1*, *sugE*, *sugE1*	IncI1, p0111, ColRNAI
ARS-CC9581	B	69	69	D	*blaCTX-M-15*, *blaTEM-1B*, *qnrS1*, *aph(3’’)-Ib*, *aph(6)-Id*, *sul2*, *tet(A)*		* *	*sugE*	IncFIB(AP001918), IncFIA, IncI1, IncFIC(FII), IncY, Col156
ARS-CC9594	C	69	69	D	*aph(3’)-Ic*, *blaCMY-2*, *blaCTX-M-27*, *blaTEM-1B*, *aph(3’’)-Ib*, *aph(6)-Id*, *tet(B)*		* *	*sugE*, *sugE1*	IncFIA, IncI1, IncFIB(AP001918), IncFII, IncFII(pCoo),
ARS-CC9607	C	69	69	D	*aadA1*, *aph(3’)-Ic*, *blaCMY-2*, *blaTEM-1B*, *catA1*, *dfrA1*, *aph(3’’)-Ib*, *aph(6)-Id*, *sul1*, *sul2*, *tet(B)*		*merA*	*qacEΔ1*, *sugE*, *sugE1*	IncFIA, IncFIB(AP001918), IncFII(pHN7A8), IncFII(pCoo), p0111
ARS-CC9610	B	69	69	D	*aph(3’)-Ic*, *blaTEM-1B*, *aph(3’’)-Ib*, *aph(6)-Id*, *sul2*, *tet(B)*		* *	*sugE*	IncFIA, IncI1, IncFIB(AP001918), IncFII(pCoo), IncY, ColRNAI, Col(MG828)
ARS-CC9628	C	69	69	D	*aadA1*, *aph(3’)-Ic*, *dfrA1*, *floR*, *aph(3’’)-Ib*, *aph(6)-Id*, *sul2*, *tet(B)*		* *	*sugE*	IncFIA, IncFIB(AP001918), IncFII(pCoo), ColRNAI
ARS-CC9653	C	69	69	D	*aph(3’)-Ic*, *blaCMY-2*, *aph(3’’)-Ib*, *aph(6)-Id*, *sul2*, *tet(B)*		* *	*sugE*	IncFIA, IncFIB(AP001918), IncFII(pCoo), IncY, IncB/O/K/Z, ColRNAI
ARS-CC9601	C	349	349	D	*aac(3)-IId*, *aadA1*, *aadA5*, *aph(3’)-Ic*, *blaCMY-2*, *blaCTX-M-27*, *blaOXA-1*, *blaTEM-1B*, *catA1*, *dfrA17*, *aph(3’’)-Ib*, *aph(6)-Id*, *sul1*, *sul2*, *tet(B)*		* *	*qacEΔ1*, *sugE*, *sugE1*	IncFIA, IncI1, IncFIB(AP001918), IncFII, IncFII(pCoo),
ARS-CC9625	B	362		D	*aph(3’)-Ia*, *blaCTX-M-15*, *blaTEM-1B*, *catA1*, *aph(3’’)-Ib*, *aph(6)-Id*, *sul2*, *tet(A)*		*merA*	*sugE*	IncQ1
ARS-CC9590	C	57	350	E	*aac(3)-VIa*, *aadA24*, *aph(3’)-Ia*, *blaCMY-2*, *dfrA1*, *floR*, *aph(3’’)-Ib*, *aph(6)-Id*, *sul2*, *tet(A)*, *tet(B)*	*ampC*-promoter (g.-32T>A), *gyrA* (p.S83L), *gyrA* (p.D87N)	*merA*	*qacEΔ1*, *sugE*, *sugE1*	IncFIA, IncFIB(AP001918), IncFII(pHN7A8), IncFII(pCoo), IncI2, ColRNAI, Col(MG828), IncA/C2, p0111, IncB/O/K/Z
ARS-CC9616	B	219		E	*aph(3’)-Ic*, *blaCMY-2*, *blaTEM-1B*, *aph(3’’)-Ib*, *aph(6)-Id*, *tet(B)*		* *	*sugE*, *sugE1*	IncFIA, IncFIB(AP001918), IncFII, IncFII(pCoo), IncB/O/K/Z
ARS-CC9543	K	648	648	F	*aadA2*, *blaCTX-M-124*, *blaTEM-1B*, *catA1*, *dfrA12*, *mph(A)*, *aph(3’’)-Ib*, *aph(6)-Id*, *sul1*, *sul2*, *tet(B)*		* *	*qacEΔ1*, *sugE*	IncQ1
ARS-CC9544	M	648	648	F	*aadA2*, *aph(3’)-Ia*, *blaCTX-M-124*, *blaTEM-1B*, *catA1*, *dfrA12*, *floR*, *mph(A)*, *aph(3’’)-Ib*, *aph(6)-Id*, *sul1*, *sul2*, *tet(A)*		*merA*	*qacEΔ1*, *sugE*	IncFII, IncFIB(AP001918), IncFIA, IncX1, IncQ1
ARS-CC9589	B	648	648	F	*aph(3’)-Ic*, *blaTEM-1B*, *aph(3’’)-Ib*, *aph(6)-Id*, *sul2*, *tet(B)*	*ampC*-promoter (g.-42C>T)	* *	*sugE*	IncFII(pRSB107), IncFIA, IncFIB(AP001918), IncFII(pCoo),
ARS-CC11293	K	457		F	*aadA22*, *aph(3’)-Ia*, *blaCMY-2*, *blaCTX-M-55*, *blaTEM-1B*, *catA2*, *dfrA14*, *floR*, *lnu(F)*, *aph(3’’)-Ib*, *aph(6)-Id*, *sul2*, *sul3*, *tet(A)*	*gyrA* (p.S83L), *gyrA* (p.D87Y)	* *	*sugE*, *sugE1*	IncFIC(FII), IncI1, IncFIB(AP001918), IncY
ARS-CC11348	I	117		G	*aadA1*, *blaCMY-2*, *sul1*		*merA*	*sugE*, *sugE1*	IncFII(pRSB107), IncFII, IncI1, IncFIB(AP001918), ColRNAI, Col156, Col(MG828)
ARS-CC9597	C	117		G	*aadA1*, *aph(3’)-Ic*, *blaCMY-2*, *sul1*, *tet(A)*		*merA*	*qacEΔ1*, *sugE*, *sugE1*	IncFII(29), IncI1, IncFIB(AP001918), ColRNAI, Col156, Col(MG828)
ARS-CC9602	C	117		G	*aac(3)-VIa*, *aadA1*, *aph(3’)-Ic*, *blaCMY-2*, *blaTEM-1C*, *aph(3’’)-Ib*, *aph(6)-Id*, *sul2*, *tet(A)*, *tet(B)*		* *	*sugE*, *sugE1*	IncHI2, IncHI2A, IncFIC(FII), IncI1, IncFIB(AP001918), p0111, ColRNAI, Col156, Col(MG828)
ARS-CC9635	C	117		G	*aadA5*, *blaTEM-1B*, *dfrA17*, *aph(3’’)-Ib*, *aph(6)-Id*, *sul2*, *tet(A)*		* *	*sugE*	IncFII, IncI1, ColRNAI, Col(MG828)
ARS-CC11349	M	3042		Clade I	*blaCMY-2*, *dfrA8*, *aph(3’’)-Ib*, *aph(6)-Id*, *sul2*, *tet(A)*, *tet(M)*	*parE*:p.I355T	* *	*sugE*	IncFII, IncQ1
ARS-CC11304	G	10	10	A			* *		
ARS-CC11308	G	10	10	A			* *		IncX4, ColRNAI
ARS-CC11342	G	10	10	A			*silABC*, *pcoABCDRSE*	*sugE*	
ARS-CC11343	G	10	10	A			*silABC*, *pcoABCDRSE*	*sugE*	
ARS-CC11324	F	730		A			* *	*sugE*	IncFIB(AP001918), IncFII(pSE11), IncFIC(FII), ColRNAI
ARS-CC11294	B	1718		A			* *	*sugE*	
ARS-CC11295	B	21	29	B1			* *	*sugE*	IncFIB(AP001918), IncY, IncB/O/K/Z
ARS-CC11306	G	21	29	B1			* *		IncFIB(AP001918), p0111, ColRNAI, IncB/O/K/Z
ARS-CC9609	B	29	29	B1			* *	*sugE*	IncFII, IncFIB(AP001918),
ARS-CC11325	F	765	29	B1			* *	*sugE*	IncFII(pSE11),
ARS-CC11296	B	162	469	B1			* *	*sugE*	IncFIA(HI1), IncFIB(pB171), IncFII(pCoo), IncI2
ARS-CC9621	B	173		B1			* *	*sugE*	IncFIB(AP001918), ColRNAI, Col156, IncB/O/K/Z
ARS-CC11305	G	300		B1			* *		IncFIB(AP001918),
ARS-CC9636	C	300		B1			* *	*sugE*	IncFIB(AP001918), ColRNAI
ARS-CC9631	C	937		B1			* *	*sugE*	IncFII, IncFIB(AP001918), IncY, Col156, ColRNAI
ARS-CC9613	B	4038		B1			* *	*sugE*	IncFIA, IncFIB(AP001918), IncFII(29), IncX1
ARS-CC11303	E	2570		D			* *		
ARS-CC11314	I	4197		E			* *		
ARS-CC9623	B	657		G			* *	*sugE*	IncFIB(AP001918), IncB/O/K/Z, ColRNAI

In total, there were 673 antimicrobial resistance genes (ARGs) detected, including 52 unique ARGs, among the 66 MDR isolates (Table 1). These ARGs conferred resistance to aminoglycosides (227 genes, 33% of detected ARGs), ß-lactams (102, 15%), sulfonamides (102, 15%), tetracyclines (97, 14%), phenicols (55, 8%), trimethoprim (51, 7%), macrolides (31, 4%), lincosamide (7, 1%), and quinolones (1, 0.1%). ARGs conferring resistance to colistin, fosfomycin, fusidic acid, glycopeptides, nitroimidazole, oxazolidinone, and rifampicin were not detected in any of the genomes. Among the isolates, the five most frequently detected ARGs were *sul2* (sulfonamide resistant dihydropteroate synthase), *aph(3’’)-Ib* (aminoglycoside phosphotransferase), *aph(6)-Id* (aminoglycoside phosphotransferase), *sul1* (sulfonamide resistant dihydropteroate synthase), and *tet(A)* (tetracycline efflux pump), and they were detected in 78%, 77%, 77%, 63%, and 63% of MDR isolates, respectively. Sulfonamide-resistant dihydropteroate synthases *sul2* and *sul1* represented 51% and 41% of the three sulfonamide ARGs detected. Aminoglycoside phosphotransferases *aph(3’’)-Ib* and *aph(6)-Id* each made up 23% of the 15 aminoglycoside ARG types detected. ß-lactamases *bla*_TEM-1B_, *bla*_CMY-2_, and *bla*_CTX-M-15_ represented 35%, 27%, and 15% of the 11 detected ß-lactamases. Tetracycline efflux pumps *tet(A)* and *tet(B)* comprised 43% and 33% of the four detected tetracycline ARGs. Macrolide 2’-phosphotransferase I *mph(A)* represented 84% of the detected macrolide ARGs. The genes *aac(6’)Ib-cr* and *qnrS1* were the only detected fluoroquinolone/quinolone ARGs. Multiple genes conferring resistance to antibiotics of human health significance were detected, including ß-lactamases *bla*_CMY-2_ and *bla*_CTX-M_ (variants 1, 15, 27, 55, 124), macrolide resistance genes *mph(A)* and *erm(B)*, fluoroquinolone/aminoglycoside resistance gene *aac(6’)Ib-cr*, and quinolone resistance gene *qnrS1*.

Metal and biocide resistance genes were detected in many isolates (Table 1). Among the MDR genomes, 25% encoded *merA* which confers resistance to mercury, 10% encoded *silABC* silver transport system, 9% encoded the *pcoABCDRSE* copper detoxification system, and one isolate encoded *arsRDABC* conferring resistance to arsenite. BRGs were also identified in a higher percentage of MDR isolates than were MRGs. In total, 50% of the MDR genomes encoded quaternary ammonium (QAC) disinfectant resistance gene *qacEΔ1*, while 80% encoded *sugE* and 27% encoded *sugE1*. Biocide resistance genes *oqxA* or *oqxB* were not detected in any of the genomes.

**Fig 2:**
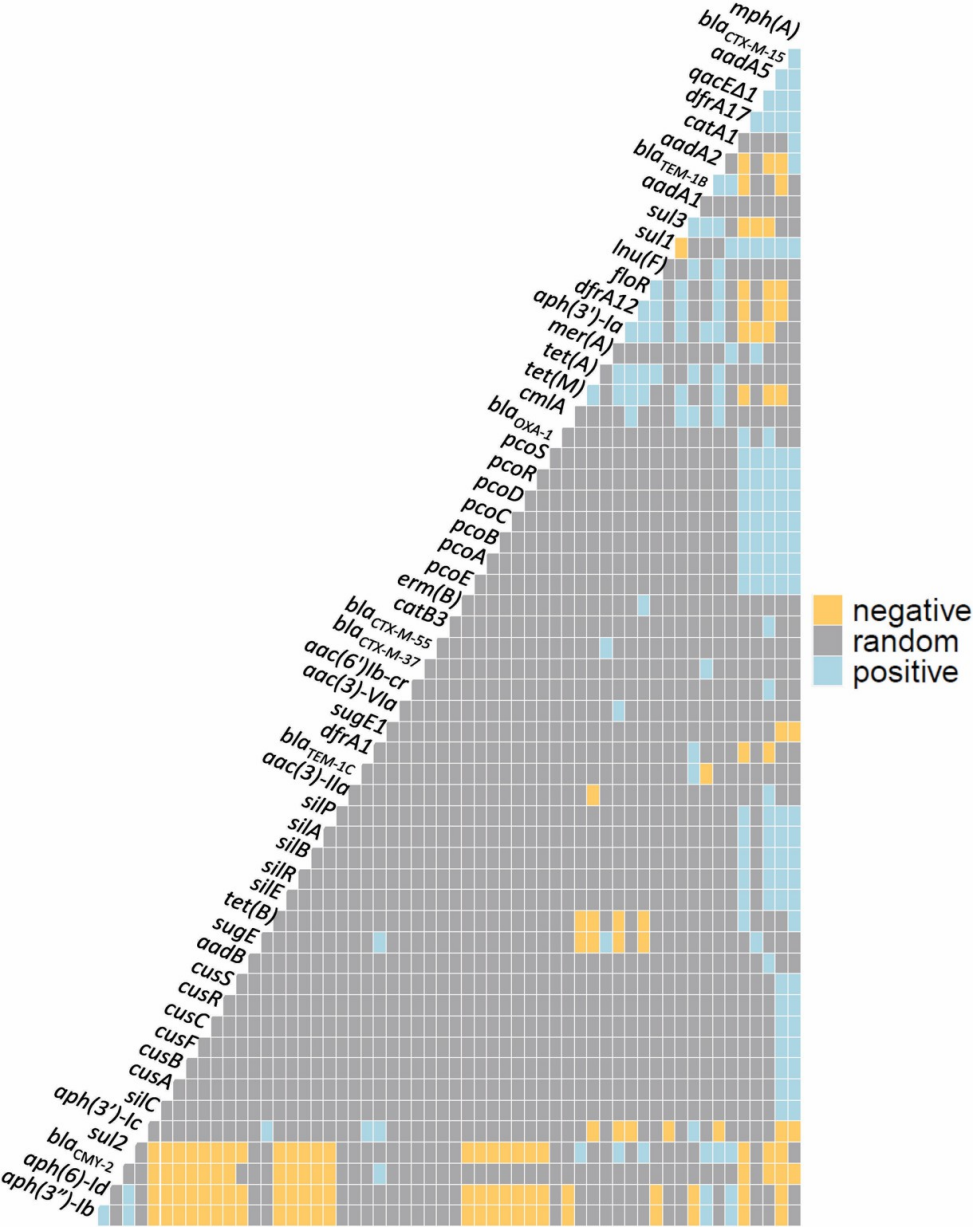
Co-occurrence matrix of antimicrobial, metal, and biocide resistance genes that show a negative, random, and positive cooccurrence with each other.

Statistically significant co-occurrence of resistance genes was detected as 160 positive co-occurrences (Fig 2). Several ARGs had positive co-occurrence with multiple resistance genes; *mph(A)*, *sul1*, *dfrA17*, *aadA5*, and *bla*_CTX-M-15_ frequently co-occurred. Silver (*sil*) and copper resistance genes (*pco*) had a positive cooccurrence with *mph(A)*, *dfrA17*, *aadA5*, and *bla*_CTX-M-15_, but negative cooccurrences with *sul2*, *aph(3’’)-Ib*, and *aph(6)-Id*. BRG *qacEΔ1* had a positive cooccurrence with *mph(A)*, *sul1*, *dfrA17*, *aadA5*, *bla*_CTX-M-15_, *pcoABCDRSE*, *merA*, and *sugE1*.

**Fig 3:**
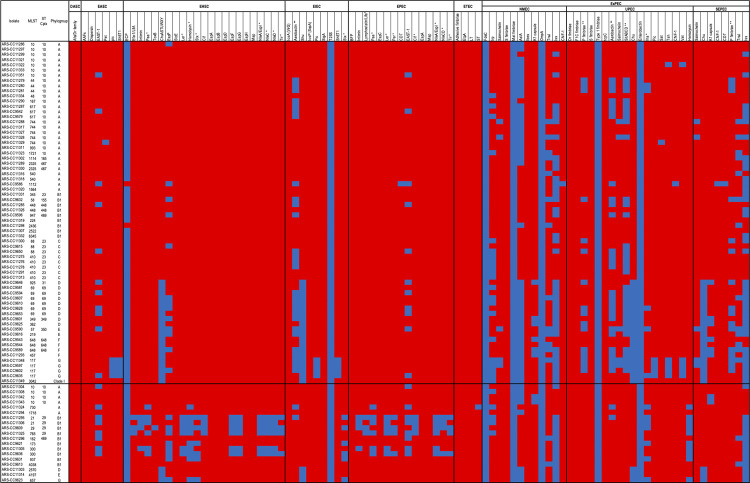
Virulence factors detected in MDR and susceptible isolates. Blue box = present. Red box = absent. * = more frequently detected in susceptible isolates. ** = more frequently detected in MDR isolates.

VFs were detected in every isolate, although the number of VFs per genome was highly variable and ranged between 1 and 48 (median = 10, mean = 15.5) (Fig 3). None of the MDR genomes encoded intimin (*eae*), translocated intimin receptor (*tir*), heat labile toxin (*eltAB*), heat stable toxin (*estlA*), the bundle forming pilus (*bfp*), or Shiga-toxins (*stx*). A single MDR isolate encoded a sequence that aligned with approximately 30% of *stx2A* at 100% similarity but was not identified as *stx2A*-positive by the VirulenceFinder tool. In total, very few VFs associated with enteroaggregative *E*. *coli* (EAEC), diffusely adherent *E*. *coli* (DAEC), enterohemorrhagic *E*. *coli* (EHEC), enteroinvasive *E*. *coli* (EIEC), enteropathogenic *E*. *coli* (EPEC), or enterotoxigenic *E*. *coli* (ETEC) were identified among the MDR isolates (Fig 3). However, VFs such as the EAEC heat-stable enterotoxin 1 (EAST1/*astA*), *Shigella* enterotoxin 1 (ShET1 or *setAB*), and *cdt* (cytolethal distending toxin) were detected in some isolates (Fig 3). There were multiple genomes encoding virulence factors known to be involved in extra-intestinal pathogenic *E*. *coli* (ExPEC) infections. These include *pap* (P fimbriae), *iha* (iron‐regulated‐gene‐homologue adhesion), *iuc-iutA* (aerobactin synthase and receptor), *irp* (iron repressible protein), *fyuA* (yersiniabactin receptor), *iroN* (salmochelin), *chu* (heme binding protein), *sit* (iron transport), *kps* (K1 capsule), *omp* (outer membrane protein), *iss* (increased serum survival), *pic* (serine protease autotransporter), *sat* (secreted autotransporter toxin), and *vat* (vacuolating autotransporter toxin) (Fig 3).

Among the MDR isolates, 35 unique plasmid replicons were detected (Table 1). IncFII and IncFIB (both often carried on the same plasmid) were the two most frequently detected replicons, followed by ColRNAI, IncFIA, IncI1, IncQ1, Col(MG828), and IncA/C2. Since the sequencing chemistry used in this study could not result in the assembly of completely closed plasmids, a co-occurrence analysis between resistance genes, VFs, and plasmid replicons was conducted to identify which plasmids may be potential carriers of certain ARGs, MRGs, BRGs and VFs (Table 2). The IncFIB replicon was positively associated with the IncFII, IncFIA, and IncB/O/K/Z replicons, as well as four ARGs, the *sit* system (*sitABCD*), and the aerobactin (*iucABC-iutA*) operon. The IncFII replicon was similarly positively associated with the *sit* system (*sitABCD*), and aerobactin (*iucABC-iutA*) operon, as well as seven ARGs. IncFIA replicons were associated with IncFIB and IncFII replicons as well as the *sit* system, aerobactin operon, 12 ARGs, and the copper resistance operon. IncA/C2 replicons were associated with 11 ARGs. BRG *qacEΔ1* was associated with IncFIA, IncFII, and IncFIC replicons, while *sugE1* was associated with IncI1, IncQ, and IncX1 replicons.

**Table 2:** ARGs, BRGs, MRGs, and VFs that co-occur with plasmid replicons identified in the study isolates.

Plasmid Replicon	ARG/BRG/MRG/VF ^A^	ARG/BRG/MRG/VF Incidence	Observed Co-occurences	Probability of Co-occurrence	Expected Co-occurences	P-value ^B^
IncA/C2	*floR*	30	16	0.066	5.6	< 0.0001
IncA/C2	*tet(M)*	22	11	0.049	4.1	< 0.0001
IncA/C2	*dfrA1*	8	4	0.018	1.5	< 0.05
IncA/C2	*aph(3’)-Ia*	20	9	0.044	3.8	< 0.01
IncA/C2	*bla*_CMY2_	28	11	0.062	5.3	< 0.01
IncA/C2	*tet(A)*	42	16	0.093	7.9	< 0.0001
IncA/C2	*aadA2*	19	7	0.042	3.6	< 0.05
IncA/C2	*aph(3")-Ib*	51	16	0.113	9.6	< 0.0001
IncA/C2	*aph(6)-Id*	51	16	0.113	9.6	< 0.0001
IncA/C2	*sul2*	52	16	0.115	9.8	< 0.001
IncA/C2	*sul1*	42	12	0.093	7.9	< 0.05
IncFIA	*bla*_OXA-1_	4	4	0.017	1.4	< 0.05
IncFIA	IncI2	3	3	0.012	1.1	< 0.05
IncFIA	*aac(3)-IIa*	3	3	0.012	1.1	< 0.05
IncFIA	*aac(6’)-Ib-cr*	3	3	0.012	1.1	< 0.05
IncFIA	*aadB*	3	3	0.012	1.1	< 0.05
IncFIA	*catB3*	3	3	0.012	1.1	< 0.05
IncFIA	*iutA*	30	22	0.125	10.6	< 0.0001
IncFIA	*iucC*	30	22	0.125	10.6	< 0.0001
IncFIA	*iucA*	30	22	0.125	10.6	< 0.0001
IncFIA	*bla*_CTX-M-15_	15	11	0.062	5.3	< 0.005
IncFIA	*sitD*	33	24	0.137	11.6	< 0.0001
IncFIA	*sitC*	33	24	0.137	11.6	< 0.0001
IncFIA	*sitB*	33	24	0.137	11.6	< 0.0001
IncFIA	*sitA*	33	24	0.137	11.6	< 0.0001
IncFIA	*iucB*	29	21	0.12	10.2	< 0.0001
IncFIA	*aph(3")-Ic*	18	13	0.075	6.4	< 0.01
IncFIA	*pcoE*	7	5	0.029	2.5	< 0.05
IncFIA	*pcoA*	7	5	0.029	2.5	< 0.05
IncFIA	*pcoB*	7	5	0.029	2.5	< 0.05
IncFIA	*pcoC*	7	5	0.029	2.5	< 0.05
IncFIA	*pcoD*	7	5	0.029	2.5	< 0.05
IncFIA	*pcoR*	7	5	0.029	2.5	< 0.05
IncFIA	*pcoS*	7	5	0.029	2.5	< 0.05
IncFIA	*mcmA*	16	10	0.066	5.6	< 0.05
IncFIA	*eilA*	16	10	0.066	5.6	< 0.05
IncFIA	*dfrA17*	21	13	0.087	7.4	< 0.01
IncFIA	*tet(B)*	32	19	0.133	11.3	< 0.01
IncFIA	*aadA5*	22	13	0.091	7.8	< 0.01
IncFIA	IncFII	53	29	0.22	18.7	< 0.0001
IncFIA	*qacEΔ1*	33	18	0.137	11.6	< 0.01
IncFIA	*fyuA*	23	12	0.096	8.1	< 0.05
IncFIA	IncFIB	59	29	0.245	20.8	< 0.0001
IncFIA	*sul1*	42	19	0.174	14.8	< 0.05
IncFIA	*sugE*	67	28	0.278	23.6	< 0.05
IncFIB	IncB/O/K/Z	11	11	0.09	7.6	< 0.05
IncFIB	IncFIC(FII)	10	10	0.082	6.9	< 0.05
IncFIB	Col156	9	9	0.073	6.2	< 0.05
IncFIB	*sitD*	33	32	0.269	22.9	< 0.0001
IncFIB	*sitC*	33	32	0.269	22.9	< 0.0001
IncFIB	*sitB*	33	32	0.269	22.9	< 0.0001
IncFIB	*sitA*	33	32	0.269	22.9	< 0.0001
IncFIB	IncFIA	30	29	0.245	20.8	< 0.0001
IncFIB	*iutA*	30	29	0.245	20.8	< 0.0001
IncFIB	*iucC*	30	29	0.245	20.8	< 0.0001
IncFIB	*iucA*	30	29	0.245	20.8	< 0.0001
IncFIB	*iucB*	29	28	0.237	20.1	< 0.0001
IncFIB	*bla*_CTX-M-15_	15	14	0.122	10.4	< 0.05
IncFIB	*espP*	21	19	0.171	14.6	< 0.05
IncFIB	*aadA5*	22	19	0.18	15.3	< 0.05
IncFIB	*tet(B)*	32	27	0.261	22.2	< 0.05
IncFIB	*qacEΔ1*	33	27	0.269	22.9	< 0.05
IncFIB	ColRNAI	38	31	0.31	26.4	< 0.05
IncFIB	IncFII	53	43	0.433	36.8	< 0.01
IncFIB	*sugE*	67	52	0.547	46.5	< 0.01
IncFIC(FII)	*cmlA1*	9	4	0.012	1.1	< 0.01
IncFIC(FII)	*dfrA12*	17	6	0.024	2	< 0.01
IncFIC(FII)	*aph(3")-Ia*	20	7	0.028	2.4	< 0.01
IncFIC(FII)	*aadA2*	19	6	0.026	2.2	< 0.01
IncFIC(FII)	IncX1	16	5	0.022	1.9	< 0.05
IncFIC(FII)	*IncI1*	22	6	0.03	2.6	< 0.05
IncFIC(FII)	*aadA1*	26	6	0.036	3.1	< 0.05
IncFIC(FII)	*bla*_TEM-1B_	36	8	0.05	4.2	< 0.05
IncFIC(FII)	*tet(A)*	42	8	0.058	4.9	< 0.05
IncFIC(FII)	aslA	51	9	0.071	6	< 0.05
IncFIC(FII)	IncFIB	59	10	0.082	6.9	< 0.05
IncFII	IncFIA	30	29	0.22	18.7	< 0.0001
IncFII	*traJ*	15	14	0.11	9.4	< 0.01
IncFII	*aph(3")-Ic*	18	16	0.132	11.2	< 0.01
IncFII	*sitD*	33	29	0.242	20.6	< 0.0001
IncFII	*sitC*	33	29	0.242	20.6	< 0.0001
IncFII	*sitB*	33	29	0.242	20.6	< 0.0001
IncFII	*sitA*	33	29	0.242	20.6	< 0.0001
IncFII	*iutA*	30	26	0.22	18.7	< 0.001
IncFII	*iucC*	30	26	0.22	18.7	< 0.001
IncFII	*iucA*	30	26	0.22	18.7	< 0.001
IncFII	*bla*_CTX-M-15_	15	13	0.11	9.4	< 0.05
IncFII	*iucB*	29	25	0.213	18.1	< 0.001
IncFII	*dfrA17*	21	18	0.154	13.1	< 0.01
IncFII	*qacEΔ1*	33	27	0.242	20.6	< 0.01
IncFII	*aadA5*	22	18	0.161	13.7	< 0.05
IncFII	*tet(B)*	32	26	0.235	20	< 0.01
IncFII	*sul1*	42	31	0.308	26.2	< 0.05
IncFII	IncFIB	59	43	0.433	36.8	< 0.01
IncFII	s*ugE*	67	47	0.491	41.8	< 0.01
IncI1	*set1B*	4	4	0.012	1	< 0.01
IncI1	*set1A*	4	4	0.012	1	< 0.01
IncI1	*pic*	4	4	0.012	1	< 0.01
IncI1	*vat*	5	4	0.015	1.3	< 0.05
IncI1	*tsh*	5	4	0.015	1.3	< 0.05
IncI1	*bla*_CTX-M-27_	5	4	0.015	1.3	< 0.05
IncI1	*lnuF*	7	5	0.021	1.8	< 0.05
IncI1	*ireA*	10	7	0.03	2.6	< 0.01
IncI1	*sul3*	8	5	0.024	2.1	< 0.05
IncI1	IncFIC(FII)	10	6	0.03	2.6	< 0.05
IncI1	ColMG828	17	10	0.052	4.4	< 0.01
IncI1	*sugE1*	18	10	0.055	4.7	< 0.01
IncI1	*sfaC*	16	8	0.049	4.1	< 0.05
IncI1	*papK*	16	8	0.049	4.1	< 0.05
IncI1	*papJ*	16	8	0.049	4.1	< 0.05
IncI1	*papI*	16	8	0.049	4.1	< 0.05
IncI1	*papH*	16	8	0.049	4.1	< 0.05
IncI1	*papF*	16	8	0.049	4.1	< 0.05
IncI1	*papD*	16	8	0.049	4.1	< 0.05
IncI1	*papC*	16	8	0.049	4.1	< 0.05
IncI1	*papB*	16	8	0.049	4.1	< 0.05
IncI1	*sfaX*	14	7	0.043	3.6	< 0.05
IncI1	*bla*_CMY-2_	28	13	0.085	7.2	< 0.01
IncI1	*chuY*	23	10	0.07	6	< 0.05
IncI1	*chuX*	23	10	0.07	6	< 0.05
IncI1	*chuW*	23	10	0.07	6	< 0.05
IncI1	*chuV*	23	10	0.07	6	< 0.05
IncI1	*chuU*	23	10	0.07	6	< 0.05
IncI1	*chuT*	23	10	0.07	6	< 0.05
IncI1	*chuS*	23	10	0.07	6	< 0.05
IncI1	*chuA*	23	10	0.07	6	< 0.05
IncI1	*shUY*	23	10	0.07	6	< 0.05
IncI1	*shUX*	23	10	0.07	6	< 0.05
IncI1	*shUV*	23	10	0.07	6	< 0.05
IncI1	*shUT*	23	10	0.07	6	< 0.05
IncI1	*shUS*	23	10	0.07	6	< 0.05
IncI1	*shUA*	23	10	0.07	6	< 0.05
IncI1	*aslA*	51	17	0.155	13.2	< 0.05
IncQ1	*mer(A)*	17	10	0.042	3.6	< 0.001
IncQ1	*catA1*	12	7	0.03	2.5	< 0.05
IncQ1	*tet(M)*	22	9	0.055	4.7	< 0.05
IncQ1	*qacEΔ1*	33	12	0.082	7	< 0.01
IncQ1	*bla*_TEM-1B_	36	13	0.09	7.6	< 0.01
IncQ1	*mph(A)*	26	9	0.065	5.5	< 0.05
IncQ1	*sul1*	42	14	0.105	8.9	< 0.01
IncQ1	*aph(3")-Ib*	51	16	0.127	10.8	< 0.01
IncQ1	*aph(6)-Id*	51	16	0.127	10.8	< 0.01
IncQ1	*sul2*	52	16	0.13	11	< 0.01

^A^: ARG = antibiotic resistance gene, BRG = biocide resistance gene, MRG = metal resistance gene, VF = virulence factor

^B^: Observed co-occurrences greater than what is expected by chance

Although the focus of this study was on highly drug-resistant *E*. *coli* isolates, we randomly selected a smaller subset of susceptible isolates as comparators to better evaluate the characteristics of the MDR isolates. Similar to the MDR isolates, the most common ST among the susceptible isolates was ST10. However, the most common phylogroup among these isolates was B1 (Table 1). The most frequently detected plasmid replicons were IncFIB, ColRNAI, and IncFII, which were detected in 11, 7, and 6 genomes, respectively. IncI1, IncQ1, and IncA/C2 replicons were not detected among the susceptible isolate genomes. EHEC and EPEC-associated VFs absent in MDR genomes were identified in susceptible isolates. These include *efa-1*/*lifA* (EHEC factor for adherence/lymphostatin), *eae*, *paa* (porcine attaching-effacing associated protein), *ler* (LEE encoded regulator), *hlyABCD* (hemolysin), *stx*, *cif* (cycle-inhibiting factor), *nleACD* (non-LEE-encoded effectors), and plasmid-encoded regulator (*per*) (Fig 3).

**Fig 4:**
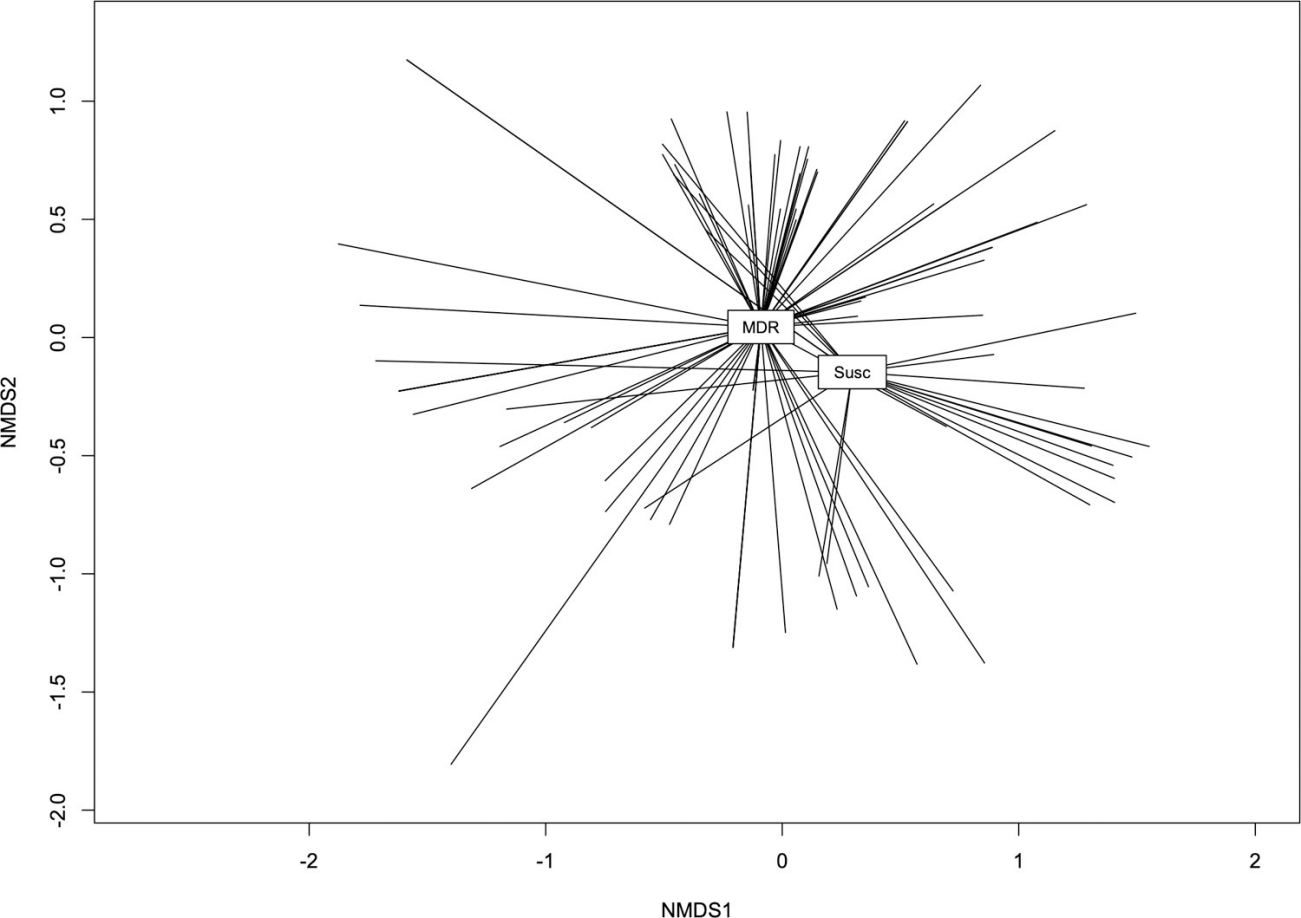
Nonmetric multidimensional scaling (NMDS) analysis of virulence gene presence/absence (Jaccard distance) with isolates grouped based on resistance gene presence (stress = 0.19) (ANOSIM R = 0.1689, P = 0.001).

Based on a nonmetric multidimensional scaling (NMDS) analysis, the virulomes of MDR isolates were somewhat different than those of susceptible isolates (ANOSIM R = 0.1698, P = 0.001) (Fig 4). Since some VFs are integral to within-host survival, we assessed if there was an association between VFs, which may confer a selective advantage in the host gut, and the MDR phenotype. We analyzed the relative abundances of these accessory genes and compared these abundances in the MDR genomes to the susceptible genomes. In total, there were 40 VFs that were differentially abundant between the two groups (Fisher’s exact test, two-tailed, q < 0.05). Of these, 22 were more abundant in susceptible genomes and 18 were more abundant in MDR genomes (Fig 3). The VFs more abundant in susceptible isolates included *stx1AB*, *stx2AB*, *ler*, *tir*, *eae*, *cif*, *paa*, *per*, *hlyABCE*, *ehxA* and *nleABCD* (q < 0.05). The VFs more abundant in MDR genomes were iron acquisition genes *sitABCD* and *iucABC-iutA* (aerobactin), and *pap* P fimbriae (q < 0.05).

**Fig 5:**
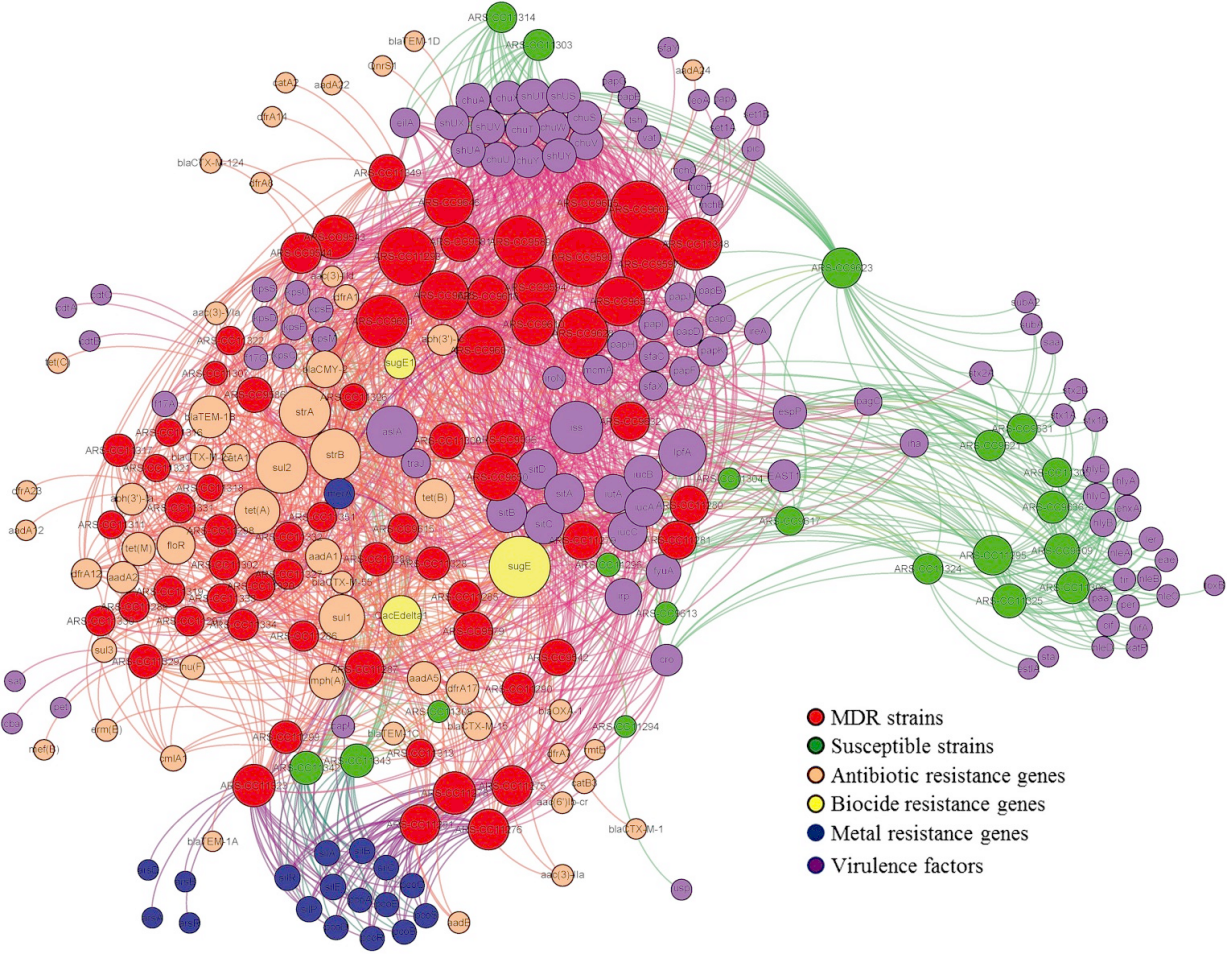
A network analysis showing the presence of resistance genes and virulence factors in all the isolates (both MDR and susceptible). The nodes are colored by the corresponding isolate and gene types. The size of each node represents the number of connected edges (degree). Each edge (curve) represents the presence of a gene in an isolate.

The presence of all resistance genes and VFs in all strains (MDR and susceptible) were visualized in a network interface (Fig 5). In this study, most of the susceptible isolates were grouped into a separate cluster from the MDR isolates in the network structure indicating variation in the resistance genes and VFs repertoires in susceptible versus MDR isolates, which is congruent with the results of the NMDS analysis (Fig 5).

Several clonal strains with high levels of genomic similarity, based on the core genome SNPs and ARGs, were isolated from different veal operations (Fig 1). Isolates from farms E and H (ARS-CC11278 and ARS-CC11291) collected 122 days apart differed by 20 SNPs and two isolates from farms B and G (ARS-CC11328 and ARS-CC11288) collected over 7 days differed by 17 SNPs.

## Discussion

Antimicrobial resistance has been well-documented in dairy and beef cattle and recent studies have demonstrated that younger calves harbor a greater abundance of resistant bacteria than older animals [10–14]. However, resistance in veal calves, which are considered a separate production class from dairy and beef calves during the Food and Drug Administration drug approval process, remains under-studied. Dairy calves raised as replacements for lactating cows and veal calves are managed differently and fed different diets. Replacement dairy calves are initially fed a diet of milk or milk replacer, followed by gradual introduction of hay and a solid calf starter. Once weaned, typically at 8–9 weeks of age, they are fed an exclusively solid feed. This phased transition to solid food assists in the development of a functional rumen. The diets of veal calves, on the other hand, typically include milk or milk replacer (made from whey and whey protein) until marketed at 16–18 weeks. Bob veal calves are fed either colostrum, waste milk, and/or milk replacer for approximately three weeks when they are sold. Although several studies have evaluated the prevalence of antimicrobial resistance on veal farms [34], none to-date have evaluated the non-ARG genetic features that co-occur with ARGs and may be associated with persistence or selection of resistance in the calf gut. Further, the public health risk posed by these bacteria remains unknown. Here we analyzed 66 MDR isolates from non-redundant veal calf fecal samples and compared these to a smaller subset of susceptible isolates with the aim of further understanding the diversity of MDR strains shed by these animals, as well as the genomic features that may be responsible for their persistence in young calves.

### High genotypic diversity with an observed predominant phylogroup and genotype

Results of this analysis demonstrate that there is a high level of diversity among MDR *E*. *coli* isolated from veal calf feces, but the group was dominated by phylogroup A-ST Cplx 10 strains (33% of all isolates). These data suggest that the MDR and susceptible strains from veal calf feces, in general, are associated with different lineages of *E*. *coli*. It appears that MDR *E*. *coli* shed in veal calf feces are more likely to be phylogroup A, while susceptible isolates are more likely to be B1. Currently it is unknown if certain phylogroups are more likely than others to acquire transferrable resistance, but previous studies have described this phenomenon, albeit some identified similar trends as observed in this study (group A strains having a high level of resistance), while others showed that different groups are more likely to be resistant than phylogroup A [35–40]. These studies characterized *E*. *coli* from a variety of non-bovine matrices and isolates from studies in which *E*. *coli* was recovered from feces were similar in phylogroup distribution to those presented here [41, 42]. Studies focused on bovine feces indicate that randomly selected generic *E*. *coli* are predominantly group B1 [43–46], while extended spectrum β-lactamase (ESBL)-producing *E*. *coli* were more likely to be phylogroup A [47], the latter being consistent with our results.

### ExPEC-associated sequence types repeatedly identified among MDR isolates

The predominant ST among the MDR and susceptible isolates was A-ST10, which is a “globally distributed” ST that is commonly isolated from a wide diversity of hosts, environments, and regions [48]. It is therefore not surprising that A-ST10 is common among *E*. *coli* from veal calf feces. More than half of the MDR isolates were identified as STs that are frequently isolated from human infections, including gastrointestinal and extra-intestinal infections. The pandemic ST131, which is the current leading cause of ExPEC infections globally, was not detected among any of the isolates, but ST69, ST410, ST117, ST88, ST617, ST648, ST10, ST58, and ST167, which are among the leading causes of non-ST131 ExPEC infections globally were all identified repeatedly, except for ST58 and ST167, which were identified once each [49–52]. A significant number of the isolates encoded VFs involved in ExPEC infections, such as *fyuA* (yersiniabactin), *sit* operon (Sit system), *iucABC-iutA* (aerobactin), *chuA* (heme binding protein), and *pap* operon (P fimbriae), which were particularly abundant in ST69, ST117, ST410, and ST648 genomes. Of particular interest, of the 33 isolates identified as ExPEC-associated STs, 27 were *bla*_CTX-M_-encoding strains, and 26 encoded the azithromycin resistance gene *mph(A)*, indicating that these potential ExPEC strains encoded resistance to antibiotics of human clinical significance.

Based on these data, there is an appreciable prevalence of ExPEC-associated STs among the MDR fecal *E*. *coli* isolated from veal calves. Previous studies have identified poultry as a significant reservoir of ExPEC isolates causing human bladder infections [50, 53–55], and results of this study indicate that veal calves may harbor similar strains. However, this study only takes into account the STs and VFs of these isolates and does not definitively identify them as pathogens. Further, more research needs to be conducted to evaluate the abundance of potential ExPEC strains in relation to the total *E*. *coli* population in the veal calf gut.

### ARGs, MRGs, BRGs, and their co-occurrence

The most frequently observed antimicrobial class to which ARGs were identified were aminoglycosides, β-lactams, sulfonamides, and tetracyclines, in decreasing order of frequency. Antimicrobial usage data on these operations were not available and national data on antimicrobial usage on veal operations is lacking, unlike dairy and beef calves for which these data have been periodically tabulated. Veal calves are considered a separate production class from dairy and beef steer calves during the FDA drug approval process, so antimicrobial usage in these animals cannot be accurately extrapolated to usage in veal calves. Currently, aminoglycosides (streptomycin), β-lactams (ampicillin and amoxicillin), sulfonamides (sulfabromomethazine, sulfamethazine, sulfaethoxypyridazine, and sulfamethazine), bacitracin, and tetracyclines are approved for oral administration in veal calves in the United States [56, 57].

Oxytetracycline and chlortetracycline have been included in scour (diarrhea) medication and supplemented in milk replacers fed to calves and may, in part, select for bacteria encoding resistance to these antibiotics. β-lactams, specifically ampicillin and amoxicillin, can be administered orally and intramuscularly for the treatment of bacterial enteritis and bovine respiratory disease (BRD), a significant cause of morbidity and mortality in calves and leads to considerable economic losses. Tulathromycin (macrolide) (subcutaneous administration), and ceftiofur (β-lactam, veterinary cephalosporin) (subcutaneous or intramuscular administration) can also be used for the treatment of BRD. Intramuscular administration of ceftiofur and florfenicol in dairy calves has been associated with a transient increase in resistant fecal *E*. *coli* [58]. Neomycin, an aminoglycoside that has been used to prevent scours in dairy calves, is not approved for oral administration in veal calves. What is not known is how historical use of antimicrobials within the birth herd (where neomycin is approved for use in replacement calves) can influence the presence and types of resistance carried by in the veal calves after they are moved from the source herd to the veal farm. These neonatal exposures should be considered a potential source of resistance that may remain within the veal calf gut after transitioning to a veal farm.

Some of the most common resistance genes among the MDR isolates confer resistance to antimicrobials approved for use in veal calves [59]. All but one MDR isolate encoded tetracycline resistance genes. β-lactamases, sulfonamide resistance genes, and aminoglycoside resistance genes were detected in most MDR isolates, and the macrolide resistance gene *mph(A)* was detected in a considerable number of isolates.

In addition to direct treatment with antimicrobials, calves can be exposed to antimicrobial residues in colostrum from cows treated with intramammary antibiotics at the time of dry-off (initiation of break from lactation) for mastitis treatment and prevention. Dairy operations often treat mastitis with first and third generation cephalosporins (β-lactams) and calves are sometimes fed unsaleable waste milk containing antimicrobial residues from treated lactating cows [60]. Since resistance genes are often co-located on mobile elements, exposure to one antimicrobial may select for multiple resistance genes [61]. For example, based on the genetic co-occurrence data from isolates in this study, exposure to neomycin or oxytetracycline could potentially select for trimethoprim (*dfrA12*), phenicol (*floR*), and/or sulfonamide (*sul2 and sul3*) resistance genes.

The co-occurrence of metal and biocide resistance genes with antibiotic resistance genes is notable and has been identified previously [10, 62–65]. Our isolate genomics results confirm the metagenomic analysis of Liu et al. [10], which showed a similar relationship in the metagenomes of dairy calf feces. Our analysis confirmed that MRGs and BRGs are associated with some ARGs, but there is also evidence of a negative cooccurrence between all metal resistance genes and the most frequently identified antibiotic resistance genes (*sul2*, *aph(3’’)-Ib*, and *aph(6)-Id*). Silver (*sil* genes) and copper (*pco* genes) resistance had the most frequent positive cooccurrence with ARGs, with some of these conferring resistance to antibiotics of human health significance such as *bla*_CTX-M-15_ (ESBL) and *mphA* (azithromycin resistance). Associations between silver and ARGs have been noted previously, although some of these are not consistent with our findings [66, 67]. Congruent with our results, silver resistance was found to be positively associated with *bla*_CTX-M_ in *E*. *coli* by Sütterlin et al. [68]. However, this was only observed with CTX-M-15 in our analysis. Copper is present in animal feeds, including colostrum, milk, milk replacer, and calf starter, and the positive co-occurrence between *pco* genes and *bla*_CTX-M-15_ and *mphA* indicates a potential selection for ARGs due to this dietary component. A positive co-occurrence between quaternary ammonium compound (QAC) resistance gene *qacEΔ* and *bla*_CTX-M-15_ and *mphA* was also observed, as well as between QAC-resistance gene *sugE1* and the ESBL gene *bla*_CMY-2_. QACs are among some of the antiseptics and have been used on farms for cleaning surfaces and equipment. It is unknown if QACs were used on these veal operations, but exposure of the dams to QACs prior to calving could potentially result in exposure of the calves to these compounds, or transmission of QAC-resistant bacteria from dam to calf.

Among these *E*. *coli* isolates there was a notable occurrence of genes conferring resistance to antibiotics of public health significance such as CTX-M, as well as quinolone resistance gene *qnrS1*, aminoglycoside and fluoroquinolone resistance gene *aac(6’)Ib-cr*, and azithromycin resistance gene *mph(A)*. Extended-spectrum β-lactamases (ESBL) are the most common proteins responsible for β-lactam resistance, and *E*. *coli* that harbor these genes are typically resistant to extended spectrum cephalosporins and monobactams. From a public health perspective, this is significant since β-lactams are among the most frequently prescribed antimicrobials globally, and ESBL-producing Enterobacteriaceae are considered a serious public health threat by the Centers for Disease Control (CDC) [https://www.cdc.gov/drugresistance/pdf/threats-report/2019-ar-threats-report-508.pdf]. The ESBLs identified in these genomes (*bla*_CTX-M-_and and *bla*_CMY_) are particularly notable since they are known to confer resistance to the 3^rd^ generation cephalosporin, ceftazidime, ceftriaxone, cefotaxime and the 4^th^ generation extended spectrum penicillin/β-lactamase inhibitor piperacillin/tazobactam [World Health Organization Essential Medicine Watch Group Antimicrobials, ttps://apps.who.int/iris/rest/bitstreams/1237479/retrieve]. Resistance to β-lactams has been increasing worldwide and CTX-M lactamases are among the most prevalent ESBLs in human infections. *bla*_CTX-M-1_ and *bla*_CTX-M-15_ are globally distributed. *bla*_CTX-M-15_ is notable because it is associated with pandemic ST131, but in these veal isolates it is mainly associated with A-ST10 Cplx strains. CTX-M genes were identified in strains with ExPEC VFs that were also STs associated with ExPEC infections (ST69, ST410, ST617, ST648, and ST167). The presence of plasmid-mediated quinolone resistance (PMQR) genes *aac(6’)Ib-cr* and *qnrS1* is significant as fluoroquinolones comprise a group of broad spectrum antibiotics of critical importance in animal and human health. Both of these genes increase the quinolone minimum inhibitory concentration (MIC) which gives these strains a competitive advantage in the presence of a fluoroquinolone challenge [69]. These genes were identified in ST69, ST617, and ST167 isolates that also encoded ExPEC VFs. Azithromycin has been historically used to treat Gram-positive infections but has shown promise as an alternative to treat infections with *Enterobacteriaceae* that may be resistant to other commonly used therapeutics [70]. Therefore, resistance to this antibiotic is potentially an emerging public health threat’ and should be closely monitored.

### Virulome differences and VFs associated with MDR and susceptible genotypes

On average, the virulomes of MDR strains and susceptible strains were also somewhat different according to the NMDS ordination analysis and the analysis of similarities test. Although it is clear that the virulence profiles of some MDR isolates are more similar to those of some susceptible isolates, it should be noted that the presence of these VFs does not confirm that the isolates are human pathogenic strains, but only indicates the potential for these strains to cause disease in humans. The health statuses of the animals were not reported and for some of these VFs their role in the pathogenesis in calves is not well-defined or are not known to cause disease in these animals. Some of these VFs are also known, or presumed, to enhance colonization of the mammalian gut, and therefore act as fitness factors that may confer a competitive advantage in the calf gut, regardless of disease outcome for the animal. Interestingly, our analysis indicated that *stx1AB* and *stx2AB* were enriched in the susceptible isolates when compared with their presence in the MDR isolates. Similarly, *eae* and *tir*, both located on the locus of enterocyte effacement (LEE) and involved in adherence to the human small intestine wall in EPEC and EHEC, were enriched in the susceptible strains and absent in the MDR strains. Their presence in susceptible strains is not surprising, but their absence in the MDR isolates is noteworthy. MDR STEC are occasionally shed by cows and calves [71–73], but among the animals sampled in this study they represent an undetectable minority based on the number of isolates collected and/or sequenced. Future work should investigate any potential interplay between carriage of *stx* and LEE and the presence of ARGs, or if there are veal management practices that select against MDR STEC.

Two accessory plasmid-borne iron acquisitions systems, *sitABCD* (Sit system) and *iucABC-iutA* (aerobactin), were significantly more abundant in the MDR isolates. Iron is a common limiting factor of bacterial growth and replication and is vital for many bacterial processes [74]. These two systems are involved in scavenging extracellular iron within the host environment, most notably the human gastrointestinal system and urinary tract. In *E*. *coli*, these two systems are primarily found on IncFIB plasmids, which are known to also encode multiple ARGs. IncFIB plasmid replicons were detected in all but one MDR isolate encoding *sitABCD* and/or *iucABC-iutA* genes and has previously been shown to encode both ARGs and iron acquisition systems [75, 76].

Milk has a low iron content and milk-fed calves are at a high risk of anemia [77, 78]. We hypothesize that the low input of iron into the calf gut may, in part, select for bacterial strains that encode accessory iron scavenging systems thereby allowing these organisms to outcompete strains lacking these systems. Similar to the phenomenon of antibiotic administration selecting for bacteria encoding complementary resistance genes, low iron environments potentially select for strains with genes encoding iron acquisition systems. Since these systems are co-located on resistance gene-encoding plasmids, the low iron input to the calf gut may coincidentally select for MDR strains in the absence of antibiotic administration and may synergistically act with resistance genes as simultaneous selection pressures to select for these strains. Similarly, P fimbriae genes (*pap*), involved in binding to glycolipids of the human urinary tract epithelial cells, were more abundant in MDR than susceptible isolates, but their role in binding to young bovine intestinal cells has not been evaluated. The differential enrichment of VFs in susceptible versus resistant strains has been previously identified in human isolates, but the selection pressures driving these trends are currently unknown [79, 80]. We suggest that such differential enrichment of accessory genes in MDR isolates confers an advantage upon these strains in the calf gut.

Based on genomic comparisons, closely related strains were isolated from different veal farms. Animals on these premises were primarily acquired from auction houses or buying stations, where animals from many different farms are typically commingled, and therefore exposed to a large suite of bacteria. This could include MDR *E*. *coli*, which they could then transmit to other animals in their cohort, either by direct contact during transport to the farm or at the farm, or through intermediary means such as farm workers or fomites. There is also the potential that different calves shedding highly similar strains were born at the same dairy farms on which they were exposed to the same microbial communities, and therefore could potentially be colonized by clonal copies of MDR *E*. *coli* that are endemic in their birth herd. Individual veal calves could not be traced back to their herd of origin to investigate this possibility. The repeated isolation of highly similar strains from different sources within a highly diverse *E*. *coli* population [43] suggests they haven an enhanced ability to persist within the veal farm environment. We have previously demonstrated that clonal MDR *E*. *coli* can be isolated from different animals (and animals of different ages) on the same farm, suggesting that transmission of *E*. *coli* occurs between animals and that some MDR strains may be selected for, or persist, in the bovine gut [15]. Results of this analysis suggest that MDR *E*. *coli* have the potential to spread between animals at auction houses and on dairy farms; transmission of these bacteria can spread between farms when co-colonized calves are sold to different veal farm operations.

This study demonstrates that MDR *E*. *coli* in veal operations are highly diverse but dominated by phylogroup A/ST Cplx 10 strains. Further, a significant proportion of these MDR strains are similar to ExPEC isolates known to cause infections and many encode VFs involved in colonization and virulence outside of the human intestine, particularly in the urinary tract. The encoded VFs include iron-scavenging systems, most likely co-located with resistance genes on plasmids, that may enhance the colonization of the low-iron, milk-fed calf gut environment. This analysis also demonstrated that ARGs of human health significance and MDR *E*. *coli* strains are circulating among veal calves in the same and different farms. Although this work focused on veal calves, it has relevance outside of this production system and future work focused on antimicrobial resistance in other systems or environments should evaluate the multiplicity of factors that may influence, or be associated with, the carriage of resistant bacteria. Research aimed towards mitigating the carriage of resistance in food animal production should consider the role of management practices, not just limited to antimicrobial administration, in the carriage and maintenance of resistant organisms.

## Supporting information

S1 Table Genome sequencing statistics for *E*. *coli* strains utilized in this study. (XLSX)
